# Extracellular matrix hydrogels with fibroblast growth factor 2 containing exosomes for reconstructing skin microstructures

**DOI:** 10.1186/s12951-024-02718-8

**Published:** 2024-07-26

**Authors:** Zheng Zhou, Ziheng Bu, Shiqiang Wang, Jianing Yu, Wei Liu, Junchao Huang, Jianhai Hu, Sudan Xu, Peng Wu

**Affiliations:** 1 Department of Orthopedics, Shanghai Tenth People’s Hospital, School of Medicine, Tongji University, Shanghai, 200072 PR China; 2 Department of Geriatric, Shanghai General Hospital, Shanghai Jiao Tong University School of Medicine, Shanghai, 200080 PR China; 3 Department of Joint and Sports Medicine, Shanghai Fourth People’s Hospital, School of Medicine, Tongji University, Shanghai, 200092 PR China

**Keywords:** Exosome, Fibroblast growth factor 2, Extracellular matrix, Tissue engineering

## Abstract

Decellularized extracellular matrix hydrogel (ECM hydrogel), a natural material derived from normal tissue with unique biocompatibility properties, is widely used for tissue repair. However, there are still problems such as poor biological activity and insufficient antimicrobial property. To overcome these drawbacks, fibroblast growth factor 2 (FGF 2) containing exosome (exo^FGF 2^) was prepared to increase the biological activity. Furthermore, the antimicrobial capacity of ECM hydrogel was optimised by using copper ions as a ligand-bonded cross-linking agent. The decellularized extracellular matrix hydrogel, intricately cross-linked with copper ions through ligand bonds and loaded with FGF 2 containing exosome (exo^FGF 2^@ECM/Cu^2+^ hydrogel), has demonstrated exceptional biocompatibility and antimicrobial properties. In vitro, exo^FGF 2^@ECM/Cu^2+^ hydrogel effectively promoted cell proliferation, migration, antioxidant and inhibited bacterial growth. In vivo, the wound area of rat treated with exo^FGF 2^@ECM/Cu^2+^ hydrogels were significantly smaller than that of other groups at Day 5 (45.24% ± 3.15%), Day 10 (92.20% ± 2.31%) and Day 15 (95.22% ± 1.28%). Histological examination showed that exo^FGF 2^@ECM/Cu^2+^ hydrogels promoted angiogenesis and collagen deposition. Overall, this hydrogel has the potential to inhibit bacterial growth and effectively promote wound healing in a variety of clinical applications.

**Supplementary Information:**

The online version contains supplementary material available at 10.1186/s12951-024-02718-8.

## Introduction

The skin is the body’s first defensive line to against exogenous stimuli [1]. A compromised skin integrity may result in infection and non-healing wounds, which are life-threatening conditions [2]. Conventional cotton-based wound dressings, while providing a basic barrier, are insufficient in establishing a conducive microenvironment essential for cell growth and wound healing. Therefore, there is an urgent need for new wound dressings that combine bioactives and antimicrobial properties to accelerate wound healing.

FGF 2 is a wide-spectrum angiogenic, mitogenic and neurotrophic factor, which is an important regulator of tissue regeneration [3, 4]. Studies have demonstrated that Fibroblast Growth Factor 2 (FGF 2) possesses the remarkable ability to stimulate the proliferation of various cell types, encompassing fibroblasts, vascular endothelial cells, and neuronal cells, thereby highlighting its potential as a promising agent for skin repair and regeneration [5–7]. Nevertheless, the application of FGF 2 is hindered by its susceptibility to degradation and its limited bioavailability. Identifying an appropriate carrier could be the key to overcoming these challenges [8]. Exosomes are natural carriers that package bioactive molecules, various proteins, and RNAs to transfer information between cells and tissues. In the past decade, exosomes have emerged as novel therapeutic effectors in drug delivery and regenerative medicine [9–11]. Therefore, we believe that utilizing exosomes loaded with FGF 2 (named exo^FGF 2^) could augment their biological efficacy.

Despite the advantages of FGF 2-loaded exosomes, issues related to their short-term effects and the absence of antimicrobial properties have yet to be addressed [12]. Studies have shown that the use of hydrogel-encapsulated exosomes for the treatment of skin injuries is superior to direct exosome injection therapy [13]. Among the myriad of hydrogels, decellularized extracellular matrix hydrogels stand out for their exceptional biocompatibility, offering a promising scaffold that faciliates cell attachment, proliferation and differentiation [13]. Meanwhile, ECM hydrogel is rich in hydroxyl and amine groups, which have the potential to form ligand bonds with metal ions [14]. During the gelation process of ECM hydrogels, the incorporation of copper ions facilitates the connection of different polymer chains by ligand sites on the polymer chains, thereby forming a stable network structure (named ECM/Cu^2+^). This hydrogel is expected to be a multifunctional hydrogel with antimicrobial and anti-inflammatory properties, and is suitable as a scaffold for exosomes by being able to improve structural integrity [15].

**Scheme 1:**
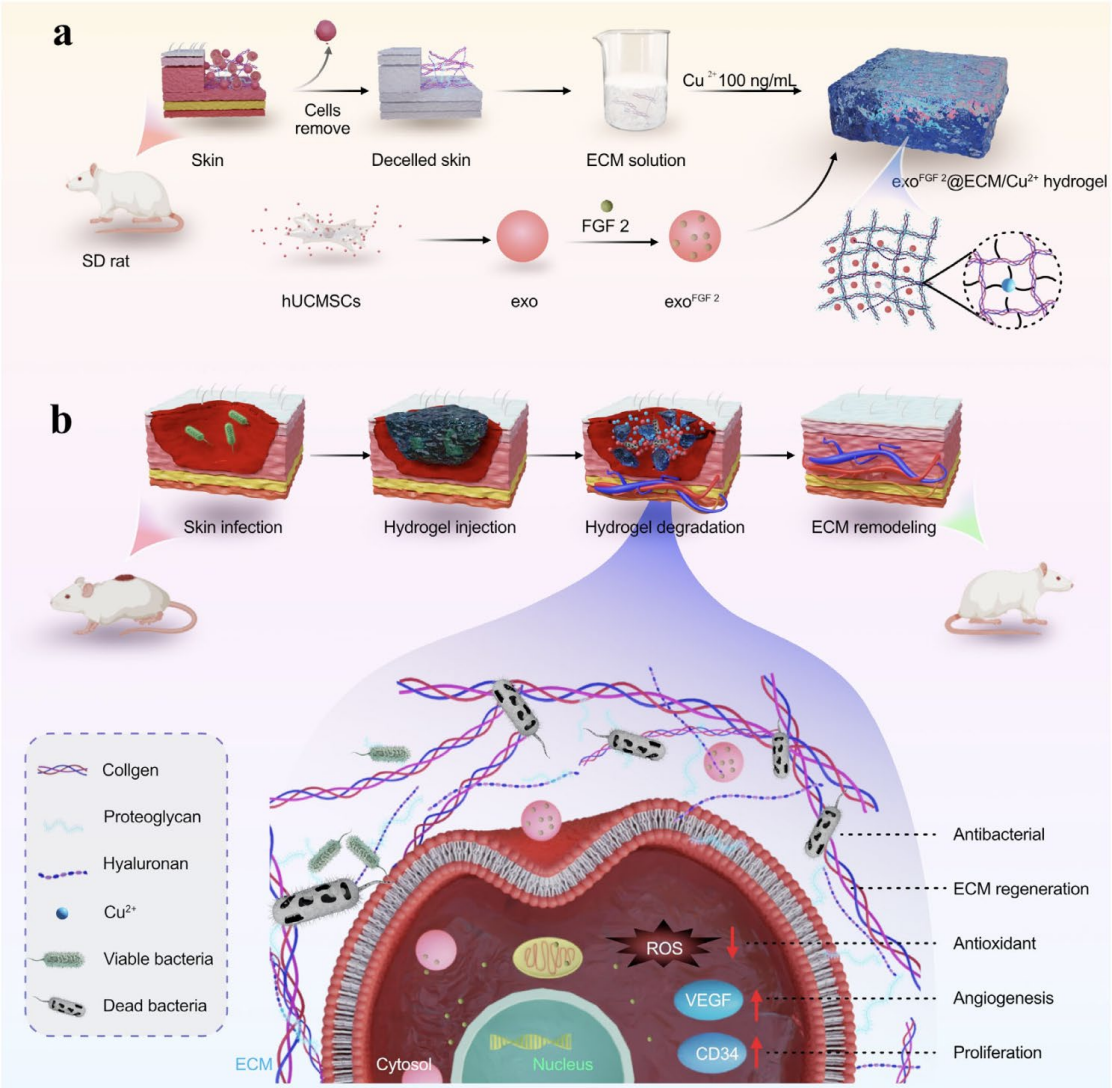
The scheme illustrates the synthetic procedure and the mechanism of skin microstructures reconstruction of the exo^FGF 2^@ECM/Cu^2+^ hydrogels. **(a)** ECM solution was obtained from rat skin and a trace amount of Cu^2+^ was used as a cross-linking agent. exo^FGF 2^ were prepared by sonicating exosomes with FGF 2. **(b)** The expected wound healing process in a mouse full-thickness skin model after application of the exo^FGF 2^@ECM/Cu^2+^ hydrogel

In this study, ECM hydrogels were obtained from rat skin, and a trace amount of Cu^2+^ was added to form ECM/Cu^2+^ hydrogels. Exosomes derived from hUCMSCs supernatant. The exo^FGF 2^ were prepared by sonicating exosomes with FGF 2. Finally, the exo^FGF 2^ was thoroughly encapsulated into ECM/Cu^2+^ hydrogels to prepare exo^FGF 2^@ECM/Cu^2+^ hydrogels. As previously mentioned, the exo^FGF 2^@ECM/Cu^2+^ solution can form hydrogels at physiological temperature (nearly 37 °C) after injection into the wound site. As the hydrogels degrade in vivo, exo^FGF 2^ and Cu^2+^ are released from hydrogels in a slow and sustained manner to maintain a high concentration at the wound site. In summary, a promising exosome-based therapeutic strategy for wound healing was proposed, which encompasses the concurrent mitigation of inflammation, facilitation of cellular migration and proliferation, stimulation of angiogenesis, and inhibition of bacterial proliferation (Scheme 1).

## Methods and materials

### Materials

hUCMSCs (Cyagen, USA, HUXUC-01101) were purchased from Cyagen Biotechnology Company. NIH/3T3 cells and HUVEC cells were purchased from the Cell Bank of the Chinese Academy of Sciences (Shanghai, China). Recombinant human basic fibroblast growth factor (FGF 2) was purchased from Nanhai Longtime Pharmaceutical Co., Ltd. (Guang Dong, China). Live/Dead kit, Thiazolyl Blue Tetrazolium Bromide (MTT), 4% Paraformaldehyde Fix Solution, Dimethyl sulfoxide (DMSO), Crystal Violet Staining Solution, Actin Tracker Red Rhodamine, DAPI were purchased from Beyotime (Shanghai, China). PKH-26 kit was purchased from Sigma (New Jersey, USA). Phosphate buffered saline (PBS, pH 7.4), Fetal Bovine Serum (FBS), Dulbecco’s modified Eagle medium (DMEM), trypsin-EDTA and penicillin-streptomycin were purchased from Thermo Fisher Scientific (Waltham, MA, USA). Trypsin, Triton-X-100, ethylenediaminetetraacetic acid (EDTA), DNase, pepsin and lysozyme were purchased from Beijing Solarbio Science & Technology Co., Ltd. (Beijing, China). Anti-vinculin-FITC antibody was purchased from Sigma-Aldrich (St Louis, MO, USA). 24-well transwell and Matrigel Matrix were purchased from Corning (New York, USA). The strains of *E. coli* (CICC 10,899) and *S. aureus* (CICC 21,601) were purchased from China Center of Industrial Culture Collection (Beijing, China). Chemicals for preparing Luria Broth (LB) agar medium were purchased from Sangon Biotech Co., Ltd (Shanghai, China).

### hUCMSCs culture and identification

The hUCMSCs were cultured in Ultra culture medium (Lonza, USA, 12–725 F) containing serum analogue Ultroser™ G (Life Science, USA, 259,509). The hUCMSCs were identified using the Human Mesenchymal Stem Cell Identification Kit stained with antibodies CD90, CD105, CD45, CD34, CD43, and HLA-DR. Fluorescence-activated cell sorter (FACS) analysis was performed using a flow cytometer and the data was analyzed using FlowJo software.

The NIH/3T3 cells and HUVECs were cultured in Dulbecco’s Modified Eagle’s Medium (DMEM) supplemented with 10% fetal bovine serum (FBS) and 1% penicillin-streptomycin. The culture was maintained in a CO_2_ incubator with a 5% level at a temperature of 37 °C.

### Isolation and identification of exo and exo^FGF 2^

Third-generation hUCMSCs 5 × 10^5^ cells/mL were cultivated on a 6-well plate. In the control group, Ultra medium containing 10% FBS was added. After 3 days of incubation, the cell medium was changed to Ultra medium with 10% FBS without exosomes for 72 h. Exosomes were then purified and collected from the collection supernatant. Again, to remove dead cells and cell debris, exosomes were centrifuged at 300 g for 10 min, 2,000 g for 10 min, and 10,000 g for 30 min at 4 °C according to a published protocol [10]. The supernatant was then transferred to a new ultracentrifuge tube and centrifuged at 100,000 g (Beckman Coulter, USA) for 70 min at 4 °C. The pellets were then resuspended in each tube with 1 mL of phosphate-buffered saline (PBS). After centrifugation at 100,000 g for 70 min, exo and exo^FGF 2^ (EVs) were obtained by resuspension with cold PBS.

These exosome samples were analyzed for DLS (Dynamic Light Scattering) to analyse particle size. Using a TEM, exosome samples were observed.

### Loading of exosomes

FGF 2 was loaded into exosomes by sonication method [16]. Briefly, the mixture of FGF 2 and exosomes were sonicated (500 volts, 2 kHz, 20% power, 6 cycles of 4 s pulse/2 s pause), cooled on ice for 2 min and then sonicated again using a Qsonica Sonicator Q700 (Fisher Scientific, Hampton, NH, USA). FGF 2 loaded exosomes were named exo^FGF 2^.

### PKH-26 staining assay

The PKH-26 (Sigma, USA, SLB6089) kit was used to label EVs according to the manufacturer’s protocol. In brief, EVs were incubated for 5 min at room temperature in the dark with 500 µL of Dilution C solution and 4 µL of PKH-26 dye solution. To halt the staining process, 500 µL of 1% bovine serum albumin (BSA) was added next. After being centrifuged at 100,000 g for 70 min, the tagged EVs were collected and resuspended in 200 µL of cold PBS. The various labelled EVs (exo and exo^FGF 2^) were adjusted to 1 × 10^9^ particles/mL, respectively, and co-cultured with NIH/3T3 cells in 48-well plates for 24 h. According to the established methodology [10], samples were washed three times with Triton X-100 (0.1%) and fixed with 4.0% PFA for 20 min. After that, the samples were incubated for 1 h with diluted Actin-Tracker Green-Rhodamine (1:100), which was diluted with a PBS solution containing 3% BSA and 0.1% Triton X-100. Finally, stained for 10 min with DAPI. With the aid of a Confocal Laser Scanning Microscope (CLSM), optical pictures were captured.

### Preparation and identification of decellularized extracellular matrix (dECM)

The dECM were made by making some changes to the method described [17]. In short, 1 cm^2^ pieces of fresh skin tissue were cut and treated with 0.25% trypsin and 1 mM EDTA in 1 PBS for 6 h at 37 °C with magnetic stirring at 300 rpm. Then, skin tissue were put in a Triton-X-100 solution (1 wt% Triton-X 100, 25 mM EDTA, 1× PBS) for 24 h at 300 rpm with magnetic stirring. Next, residual lipids were removed with 10% isopropanol for 24 h, while the Triton-X-100 and isopropanol solution were changed every 12 h. Tissues that had been treated were rinsed in 1× PBS solution for 12 h and then treated with nuclease solution (30 U/mL DNase, 10 mM MgCl_2_, 1× PBS) at 37 °C for 24 h with gentle agitation to remove all cells. The samples that had been treated with DNase were washed with PBS solution (150 rpm for 24 h) and then distilled water (150 rpm, 30 min). Then, they were sterilized for 2 h with 0.1% peroxyacetic acid in 4% ethanol, and washed several times with PBS solution and distilled water. Lastly, the dECM were dried out and put in a sealed container.

To determine the efficacy of decellularisation, histological sections were stained with H&E, Masson’s trichrome, and DAPI. The amount of remaining DNA and extracellular matrix (ECM) components such as collagen and glycosaminoglycans (GAGs) were measured [18]. dECM were digested in papain solution (125 g/mL papain, 0.1 M Na_3_PO_4_, 5 mM Na_2_EDTA, 5 mM cysteine-hydrochloric acid, pH 6.5) at 60 °C for 16 h to determine its concentration. An equal amount of local tissue was digested and utilized as a control in the same manner. Using Hoechst 33,324 test, the DNA content was measured. A fluorescence spectrophotometer (excitation wavelength: 360 nm, emission wavelength: 450 nm) was used to detect the fluorescence intensity (360 nm excitation wavelength, 450 nm emission wavelength) to determine the quantity of residual DNA in the dECM and the native tissue. Using calf thymus DNA, a standard curve for DNA was constructed and used to measure the DNA in samples. The collagen content was measured using a standard hydroxyproline test. The absorbance of the samples was measured at 560 nm and quantified using a hydroxyproline-based standard curve. The 1,9-dimethylmethylene blue solution was used to measure the quantity of sulfated glycosaminoglycans to calculate the GAGs content. The absorbance was measured using a 96-well plate at 525 nm wavelength. The chondroitin sulphate A standard curve was used to estimate the sulfated glycosaminoglycans in samples.

### Preparation of composite hydrogels

Freeze-dried 2 g of dECM were dissolved in 100 mL of a 0.5 M acetic acid solution containing 10 mg of pepsin per 100 mg of dECM and kept at room temperature for 3 days. Under cold circumstances (below 10 °C), the dECM solution’s pH was corrected to 7.4 using 10 M NaOH, and this hydrogel was named ECM.

Copper ions (100 ng/mL) were added to dECM solution. Under cold circumstances (below 10 °C), the dECM solution’s pH was corrected to 7.4 using 10 M NaOH, this hydrogel was named ECM/Cu^2+^.

exo^FGF 2^ were added to dECM solution. Under cold circumstances (below 10 °C), the dECM solution’s pH was corrected to 7.4 using 10 M NaOH. This hydrogel was named exo^FGF 2^@ECM.

Similarly, exo^FGF 2^ (10 × 10^8^ particles/mL) and copper ions (100 ng/mL) were added to dECM solution, and the pH was adjusted to about 7.4 under chilling conditions. It was named exo^FGF 2^@ECM/Cu^2+^.

### Characterization of composite hydrogels

Wet hydrogel samples were lyophilized after being frozen at -80 °C. SEM images captured the structural features and mapping hydrogels. Fourier-transformed infrared spectroscopy (FTIR, EQUINOX55, Bruker, Germany) was investigated the chemical structure and status of chemical bonds in the scaffolds within the wavelength range of 500–4000 cm^− 1^, IR spectroscopy was conducted.

### Release characteristics of bioactive substances from composite hydrogels

The bioactive components of ECM/Cu^2+^ hydrogels, exo^FGF 2^@ECM hydrogels and exo^FGF 2^@ECM/Cu^2+^ hydrogels were dispersed in 10 mL PBS by shaking at 37 °C and 200 rpm. At a preset period, 500 µL of the release media was collected and centrifuged at 7,000 rpm. The supernatant was collected for analysis and the release media was supplemented at 37 °C with an equivalent amount of fresh medium. exo^FGF 2^ were quantified using a BCA protein kit, and Cu^2+^ were assessed via inductively coupled plasma mass spectrometry (ICP-MS, PerkinElmer, USA).

### Swelling behavior test

At 37 °C, the swelling capacity of hydrogels were measured by swelling in PBS. At predetermined intervals, surface water samples were scrubbed clean with filter paper and weighed. The swelling rate (SR) was obtained according to the formula:

$${\rm{SR = }}{{{W_t} - {W_o}} \over {{W_o}}}{\rm{ \times 100\% }}$$

where *W*_t_ is the weight of the wet sample over a predetermined time interval and *W*_o_ is the initial weight of the sample.

### Mechanical characterization

Mechanical properties of those hydrogels were investigated using a uniaxial mechanical tester (Heng Yu Instrument CO., LTD. HY-940FS, Shanghai, China). The inner diameter and depth of the mold were both 1.0 cm. The accurate diameter and height of each sample were carefully recorded before testing. Those hydrogels were compressed at a constant strain rate of 5 mm/min until broken. The compressive Young’s modulus was calculated using Equation for 50% strain:

$${\text{E}}_{50{\%}}=({F}_{50\%}/S)/strain$$

Where F_50%_ was the force at 50% stress (*N*) and *S* was the cross-sectional area of the sample (m^2^). Three samples were tested for each group.

### In vitro degradation study

To measure the enzymatic degradation property of the those hydrogels, samples were placed in 5 U/mL and collagenase type I solution (Worthington, USA) and incubated at 37 °C with a speed of 60 rpm. At the desired timepoint, the hydrogel samples were removed from the degradation buffer, blotted dry and weighed. The degradation rate (DR) of the ECM hydrogel was computed by using the formula below.

$$\text{DR}=({\text{W}}_{\text{o}}-{\text{W}}_{\text{t}})/{\text{W}}_{\text{t}}\times 100\%$$

where W_t_ is the weight of the wet sample over a predetermined time interval. W_0_ is the initial weight.

### Ionic extraction preparation

Samples, including ECM, ECM/Cu^2+^ and exo^FGF 2^@ECM/Cu^2+^, prepared for making ionic extraction were sterilized with 75% ethanol for 2 h and washed three times with PBS (15 min/time). Ionic extraction of these samples was performed red according to International Standard Organization (ISO/EN) 10993-5 by directly immersing samples into DMEM with 10% FBS at 37 °C in an incubator with 5% CO_2_ for 24 h.

### Proliferation, transwell, wound healing, and tubule formation assay

Cell proliferation was assessed by the CCK-8 method. Briefly, NIH/3T3 cells were seeded at a density of 5 × 10^3^/well in 96-well plates and cultured at 37 °C in a 5% level CO_2_ incubator with a complete medium containing different EVs and different ionic extraction as described above. Cells cultured in a complete medium were set as a control group. At predetermined time points, the medium was removed and washed twice with PBS, then 100 µL of serum-free medium and 10 µL of CCK-8 solution were added to each well. Cell viability was assessed on Day 1, Day 3, and Day 5 using the CCK-8 method. Cell viability was determined according to the following formula:

$${\rm{Cell}}\,{\rm{viability = }}{{O{D_s} - O{D_b}} \over {O{D_c} - O{D_b}}}{\rm{ \times 100\% }}$$

where *OD*_*s*_, *OD*_*c*_, and *OD*_*b*_ indicate the absorbance of the sample, control, and blank respectively.

The transwell assays were performed on a 24-well transwell plate. NIH/3T3 cells were diluted to 1.0 × 10^5^ cells/mL in serum-free media before adding 200 µL to the top chamber of the 24-well transwell plate and 500 µL to the bottom chamber. 500 µL of complete medium was supplied to the bottom chamber as a control. Cells were scraped from the top chamber using a dry cotton swab after 24 h of incubation at 37 °C. Migrating cells were rinsed in PBS, fixed with 4% PFA, and stained with 0.1% crystal violet. The migrating cells were photographed using a fluorescence microscope (Leica, Germany). All samples were obtained in triplicate, and migrating cells were counted in three locations of each sample at random.

HUVECs were seeded on the Matrigel matrix to evaluate the angiogenic ability of EVs. The substrate was dispersed onto pre-cooled 24-well plates using pre-cooled pipette tips and solidified for 1 h at 37 °C. The Matrigel matrix was then inoculated with 500 µL of a 2.0 × 10^4^ cells/mL HUVECs solution containing the previously mentioned EVs and different ionic extraction, followed by 4 h incubation. Finally, microscopic images were acquired, and the total segment length was measured using the Angiogenesis Analyzer plug-in for ImageJ to evaluate tubule formation capability.

The scratch assay was performed on a 12-well plate. When the cells reached approximately 95% confluency with the extracted liquid (48 h) per well, the scratch was made by using a 200 µL pipette tip. Then the cells were inoculated with EVs and different ionic extraction for 24 h, a phase contrast microscope was used to visualize the images. The images were analyzed with Image J version 5.0.

### In vitro antibacterial activity evaluation of composite hydrogels

Gram-negative *E. coli* bacteria and Gram-positive *S. aureus* bacteria were chosen to assess the antibacterial activity. Copper ions’ antibacterial activity was evaluated using both the agar disc diffusion method and the MTT technique [19].

Copper ions were diluted with LB medium to varied concentrations and then mixed with bacterial suspensions to create a suspension containing 1 × 10^6^ CFU/mL. Bacteria treated with LB without copper ions served as the control group. After the prescribed time of incubation at 37 °C, the MTT stock solution was added to the LB medium, followed by another 4 h of incubation at 37 °C. Then, at room temperature, the formazan crystals were dissolved in DMSO, and the optical density (OD) value at 570 nm was determined using a spectrophotometer.

The liquid bacterial solution was serially diluted with sterile water to produce 1 × 10^4^, 1 × 10^5^, 1 × 10^6^, 1 × 10^7^, 1 × 10^8^, and 1 × 10^9^ diluted bacterial solutions. On bacteriological agar plates, 200 µL of diluted 1 × 10^7^, 1 × 10^8^, and 1 × 10^9^ bacteriological solutions were placed. With a sterile implement, the bacteriological solution was then spread evenly across the plate. The coated plate was inverted and incubated until the colonies have grown and can be enumerated.

To further test the antibacterial ability of EVs or different hydrogels, exo, exo^FGF 2^, ECM hydrogel, ECM/Cu^2+^ hydrogel and exo^FGF 2^@ECM/Cu^2+^ hydrogel (10 mm in diameter and 2 mm in height) were co-culture with bacterial suspension 1.0 × 10^6^ CFU/mL was incubated at 37 °C for 12, 24, 48 h. 100 µL of bacterial suspension was removed separately, washed 3 times with 0.85% saline, stained with live and dead bacterial staining kits (DMAO and EthD-III), incubated for 15 min, and observed with a fluorescence microscope (Leica, Germany). Three replicates were set up for each experiment.

### Live and death assay

The effect of exo, exo^FGF 2^ and ionic extraction of ECM, ECM/Cu^2+^ and exo^FGF 2^@ECM/Cu^2+^ on cells were tested by a live/dead assay. NIH/3T3 cells were incubated in the complete medium at an initial density of 1 × 10^4^ per well in a 96-well plate for 24 h. Then the medium was removed, exo, exo^FGF 2^and ionic extraction of ECM hydrogel, ECM/Cu^2+^ hydrogel, and exo^FGF 2^@ECM/Cu^2+^ hydrogel were added to each well. At a specified point in time, cells were washed with PBS three times, followed by adding 100 µL staining solution per well. After incubating at 37 °C for 30 min, cells were imaged under a fluorescence microscope (Leica, Germany).

### Hemolysis ratio testing

Fresh blood was harvested from the carotid arteries of SD rats by using an anticoagulant tube. Then the 4% w/v erythrocyte solution was obtained by centrifuging the blood at 1500 rpm for 15 min and resuspending the sediment with normal saline solution. The exo, exo^FGF 2^, ECM hydrogels, ECM/Cu^2+^ hydrogels and exo^FGF 2^@ECM/Cu^2+^ hydrogels were placed into a 96-well plate with 100 µL normal saline solution in each well, which was set as the experimental group. 100 µL of deionized water and saline in each well were set as positive control and negative control, respectively. A final concentration of 2% w/v was prepared by adding 100 µL of the erythrocyte suspension to each well. After further culture at 37 °C for 4 h, the 96-well plate was photographed from the side and observed by a microscope. After centrifuging at 1500 rpm for 15 min, 100 µL of supernatant was taken from each well and transferred into a new 96-well plate for taking photographs from the top side. Finally, the absorbance value at 540 nm was measured by a microplate reader (SpectraMax iD5, Molecular Devices, CA, USA) to assess haemoglobin release. The data was expressed as an average value with standard deviation (Mean ± SD, *n* = 5). The hemolysis ratio was calculated using the following equation:

$${\rm{Hemolysis }}\,{\rm{ratio = }}{{A{b_1} - A{b_2}} \over {A{b_3} - A{b_2}}}{\rm{ \times 100\% }}$$

where *Ab*_*1*_, *Ab*_*2*_, and *Ab*_*3*_ are the absorbance values of experimental, negative control, and positive control groups, respectively.

### Cell attachment and cell cycle assay

Precursor solutions for the ECM, ECM/Cu^2+^ and exo^FGF 2^@ECM/Cu^2+^ composites were filtered, applied on glass-bottomed cell culture plates at a thickness of 2 mm. Subsequent pH adjustments were meticulously executed to facilitate hydrogel formation. Hydrogels washed three times with PBS, followed by a 2-hour exposure to UV light for sterilization. After that, complete media was used to cultivate NIH/3T3 cells that had been implanted at a starting density of 1 × 10^4^ cells/well in glass jars. The cells were washed with PBS and fixed in 4% PFA for 10 min after 5 days of incubation. Rhodamine-phalloidin and anti-vinculin solution were incubated on the cells for 45 min after they had been permeabilized for 2 min with 0.2% Triton X-100 solution. DAPI was then applied for 5 min. Using a fluorescent microscope, fluorescence pictures were recorded (Manufactured by Leica in Germany).

### In vivo biocompatibility assay

18 female Sprague-Dawley (SD) rats that were 4 weeks old had exo, exo^FGF 2^, ECM hydrogels, ECM/Cu^2+^ hydrogels and exo^FGF 2^@ECM/Cu^2+^ hydrogels injected under their epidermis (*n* = 3). To assess the toxicity of these materials, major organs including the heart, liver, spleen, lung, and kidney were removed and stained with H&E. No untreated rats were used as controls. All relevant animal studies have been given the go-ahead by the Institutional Animal Care and Use Committee (IACUC).

### Full-thickness skin defect model and treatment

To create a full-thickness skin defect model, female SD rats (B & K Universal Ltd., China) were surgically incised. Shortly after receiving an intraperitoneal injection of 3% sodium pentobarbital (30 mg/kg), the rats’ back hairs were shaved off and washed with 75% alcohol. Then, on each side of each rat, two full-thickness excisional wounds (10 mm×10 mm) were created. As previously mentioned, the flaws were treated with exo, exo^FGF 2^, ECM hydrogels, ECM/Cu^2+^ hydrogels and exo^FGF 2^@ECM/Cu^2+^ hydrogels. Negative controls were not treated. On Day 5, 10, and 15 after sacrifice, wounds and surrounding healthy skin were removed and fixed in 4% PFA for further histological and immunohistochemical examination.

### Wound closure measurements

After 0, 5, 10, and 15 days, wounds were photographed using a digital camera, and ImageJ software was used to measure the wound product. The following formula was used to determine the percentage of wound closure:

$${\rm{Wound }}\,{\rm{closure = }}\left( {{\rm{1 - }}{{\rm{P}} \over {\rm{I}}}} \right){\rm{ \times 100\% }}$$

where *I* represents the original wound site and *P* represents the wound site at a specific time. For the statistical analysis, three samples from each treatment group were examined.

### Histological, immunofluorescence, and immunohistochemical evaluation

Tissues excised and fixed in PFA solution were dehydrated and embedded in paraffin, then cut to 5 μm and mounted on slides for staining. Following the manufacturer’s instructions, the sections were stained using Masson trichrome and Hematoxylin and Eosin staining kits. To assess the ability for angiogenic activity and cell proliferation, the sections were subsequently stained with anti-CD34, anti-VEGF, and DAPI antibodies, respectively. A digital microscope was used to take pictures (Leica, USA).

### Statistical analysis

Data was expressed as mean ± standard deviation (SD). Significance between groups were analyzed by Student’s t-test and one-way analysis of variance (ANOVA) using SPSS software (version 25.0, IBM). Statistical significance was set at *p < 0.05, ** *p* < 0.01, *** *p* < 0.001, ****p < 0.0001.

## Results

### Isolation and characterization of hUCMSCs, exo, exo^FGF 2^

**Fig. 1:**
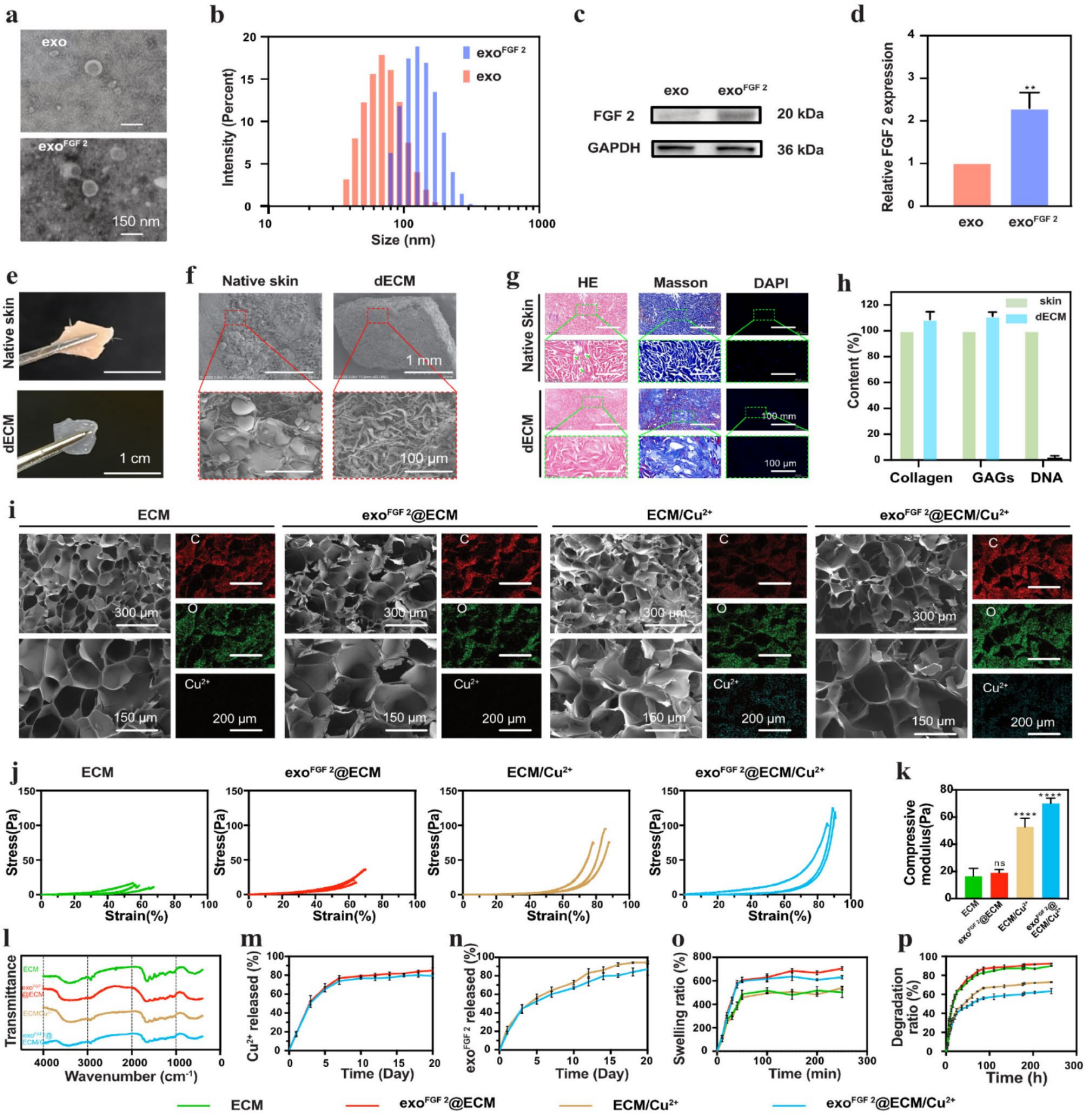
Preparation and characterization of exo^FGF 2^@ECM/Cu^2+^ hydrogels. (**a**) TEM images of exo and exo^FGF 2^. (**b**) Particle size distribution of EVs. (**c**) Western blot analyse of FGF 2 proteins expressed in EVs. (**d**) Quantification of protein expression. Error bars indicate the SD (*n =* 3). (**e**) Digital and (**f**) SEM of SD rat skin tissue before and after decellularisation. (**g**) Hematoxylin-eosin (H&E) staining, Masson’s trichrome staining, and DAPI staining of SD rat skin tissue before and after decellularisation. (**h**) Quantitative analysis of ECM solution. Error bars indicate the SD (*n* = 3). (**i**) SEM images and elemental mapping images of ECM hydrogel, exo^FGF 2^@ECM hydrogel, ECM/Cu^2+^ hydrogel, exo^FGF 2^@ECM/Cu^2+^ hydrogel. (**j**) Compressive stress–train curve of ECM hydrogel, exo^FGF 2^@ECM hydrogel, ECM/Cu^2+^ hydrogel, exo^FGF 2^@ECM/Cu^2+^ hydrogel. (**k**) The compressive Young’s modulus of ECM hydrogel, exo^FGF 2^@ECM hydrogel, ECM/Cu^2+^ hydrogel, exo^FGF 2^@ECM/Cu^2+^ hydrogel. Error bars indicate the SD (*n* = 3). (**l**) Fourier transform infrared (FTIR) spectroscopic analyses of ECM hydrogel, exo^FGF 2^@ECM hydrogel, ECM/Cu^2+^ hydrogel, exo^FGF 2^@ECM/Cu^2+^ hydrogel. (**m**) The cumulative Cu^2+^ release percentage from ECM/Cu^2+^ hydrogel and exo^FGF 2^@ECM/Cu^2+^ hydrogel. Error bars indicate the SD (*n* = 3). (**n**) The cumulative release percentage from exo^FGF 2^@ECM hydrogel and exo^FGF 2^@ECM/Cu^2+^ hydrogel (*n* = 3). (**o**) Swelling rate of ECM hydrogel, exo^FGF 2^@ECM hydrogel, ECM/Cu^2+^ hydrogel, exo^FGF 2^@ECM/Cu^2+^ hydrogel. Error bars indicate the SD (*n* = 3). (**p**) Degradation rate of ECM hydrogel, exo^FGF 2^@ECM hydrogel, ECM/Cu^2+^ hydrogel, exo^FGF 2^@ECM/Cu^2+^ hydrogel. Error bars indicate the SD (*n* = 3). Statistical differences were determined using an ANOVA with Bonferroni’s multiple comparison test (*p < 0.05, **p < 0.01, ***p < 0.001, ****p < 0.0001)

Microscopically, the morphology of hUCMSCs showed spindle-shaped cells (Fig. S1a). Using flow cytometry, the hUCMSCs surface markers CD90, CD105, CD45, CD73, CD34, and HLA-DR was identified (Fig. S1b). The TEM images show that both exo and exo^FGF 2^ have a distinct central depression (Fig. 1a) with a diameter of about 50–180 nm (Fig. 1b). According to the data from the DLS, 99% of exo and exo^FGF 2^ had diameters between 30 and 180 nm, exo^FGF 2^ was larger after drug loading compared to exo (Fig. 1b). Additionally, WB results showed a significant increase in FGF 2 levels after ultrasound loading (Fig. 1c, d). However, a small amount of FGF 2 was also detected in the exo group, possibly indicating that a small amount of FGF 2 was already present in the exo. Overall, these findings verified the effective extraction of exosomes and the successful fabrication of exo ^FGF 2^. Based on the results of the CCK8 assay, the optimal concentration of exo^FGF 2^ is 11 × 10^9^ particles/mL, a concentration that maximally promotes cell proliferation (Fig. S6).

### Evaluations of decellularization effectiveness

As depicted in (Fig. 1e), the rat skin is translucent after decellularisation. The SEM results show (Fig. 1f) that the native epidermis appeared morphologically disorganized and full of cells and fat, whereas the dECM had a porous, fibrous structure without cells. Hematoxylin-eosin staining (H&E), Masson’s trichrome staining and DAPI staining confirmed that the primary ECMs, collagen, and GAGs, were well-preserved, whereas the nucleus were scarcely discernible (Fig. 1g). Further quantitative analyses showed that collagen (114%) and GAGs (94%) were largely preserved, while DNA (98.1%) was largely removed from the native epidermis(Fig. 1h). Therefore, all results support the effectiveness of our decellularization procedure.

SEM was used to examine the microstructures of freeze-dried hydrogels. As shown in Fig. 1i, all hydrogel samples showed a typical interconnected porous structure with pore sizes of a few micrometers. By elemental mapping the distribution and ratios of Cu^2+^ in ECM/Cu^2+^ hydrogels and exo^FGF 2^@ECM/Cu^2+^ hydrogels were evenly distributed throughout the composite hydrogels.

To evaluate the mechanical properties of ECM hydrogels, ECM/Cu^2+^ hydrogels, exo^FGF 2^@ECM hydrogels and exo^FGF 2^@ECM/Cu^2+^ hydrogels. The compressive stress-strain curves are measured via a uniaxial mechanical tester. The compressive Young’s modulus of ECM hydrogels, exo^FGF 2^@ECM hydrogels, ECM/Cu^2+^ hydrogels, and exo^FGF 2^@ECM/Cu^2+^ hydrogels are determined to be 16.54 ± 3.323 Pa, 19.11 ± 1.344 Pa, 52.75 ± 3.738 Pa and 70.04 ± 2.250 Pa, respectively (Fig. 1j, k). The increase in compressive Young’s modulus is due to the introduction of Cu^2+^ into hydrogel. Using copper ions as a ligand-bonded cross-linking agent enhanced the structure of the hydrogel (*p* < 0.0001).However, the addition of exo^FGF 2^ did not affect the mechanical properties of hydrogel.

The chemical structure of the hydrogels was examined using Fourier transform infrared spectroscopy (FTIR) (Fig. 1l). The peaks located at 3423 cm^− 1^ (O-H and N-H), 1080 cm^− 1^ and 1030 cm^− 1^ (C-O), 1745 cm^− 1^ (C-O), 1543 cm^− 1^ (N-H), and 1236 cm^− 1^ (C-N), respectively, demonstrated the successful formation of hydrogels. The relatively slow release of Cu^2+^ from the ECM/Cu^2+^ hydrogels and exo^FGF 2^@ECM/Cu^2+^ hydrogels was detected within 20 days, which was effectively inhibit bacterial growth during the wound repair process (Fig. 1m). exo^FGF 2^@ECM/Cu^2+^ hydrogels and exo^FGF 2^@ECM hydrogels provide a sustained release of exo^FGF 2^ over a period of 20 days, which is sufficient to promote skin repair (Fig. 1n). The results of the swelling test showed all of the samples exhibited exceptionally high swelling rates (greater than 400%). Notably, ECM/Cu^2+^ hydrogel and exo^FGF 2^@ECM/Cu^2+^ hydrogel exhibited a lower maximal swelling rate than ECM hydrogel and exo^FGF 2^@ECM hydrogel, which may be attributable to the increased ionic strength of the ECM/Cu^2+^ hydrogel and the exo^FGF 2^@ECM/Cu^2+^ hydrogel (Fig. 1o). The results of degradation experiments showed that hydrogels containing copper ions degraded more slowly, probably because copper ions strengthened their structure. This slow degradation property may prolong the release of copper ions and exosomes for better therapeutic effect (Fig. 1p).

### Cell proliferation, migration, and tube formation

**Fig. 2:**
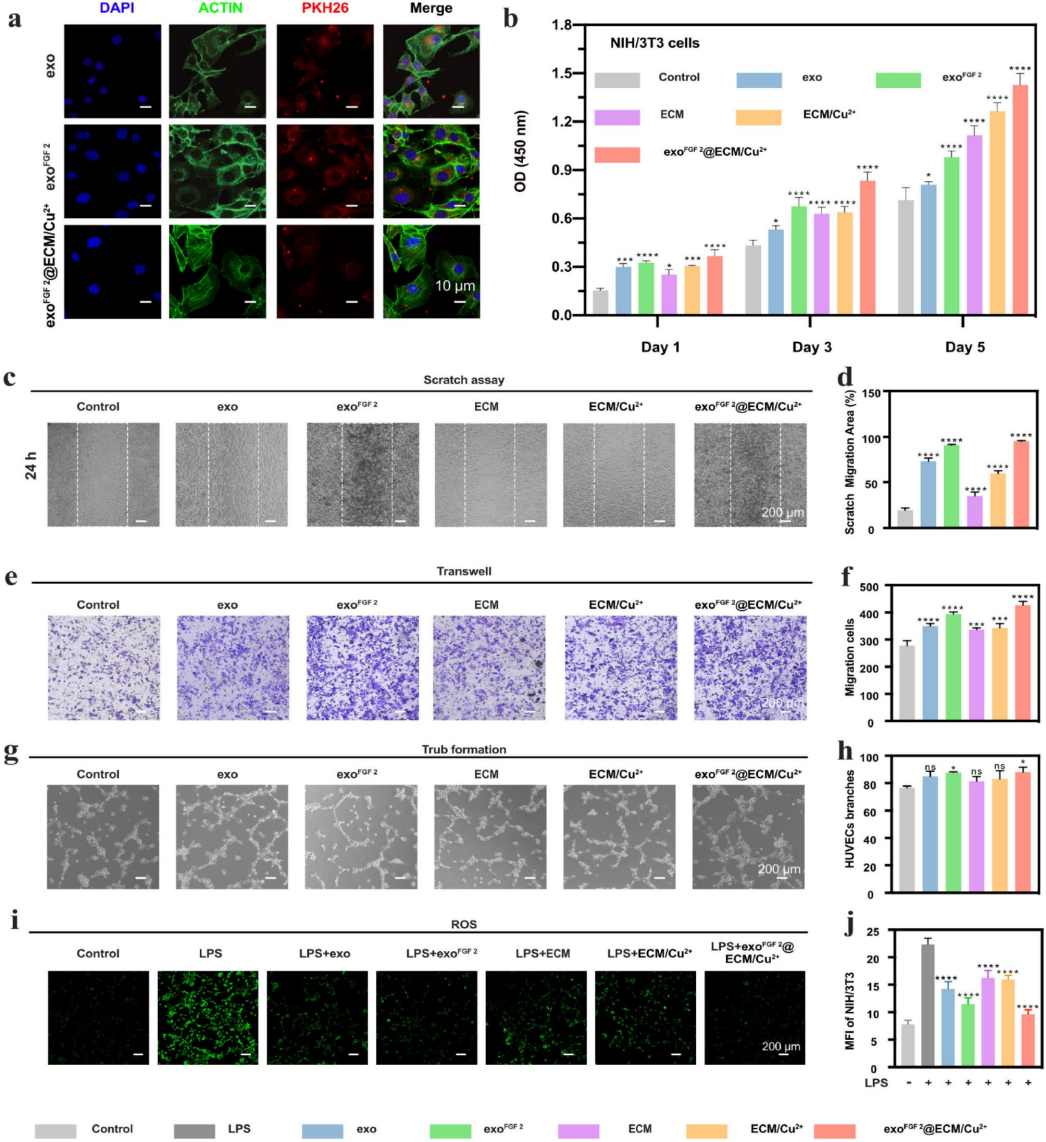
In vitro evaluation of exo, exo^FGF 2^, ECM hydrogel, ECM/Cu^2+^hydrogel and exo^FGF 2^@ECM/Cu^2+^ hydrogel. (**a**) Representative fluorescence micrograph of PKH-26 (red)-labelled EVs internalized by NIH/3T3 cells. (**b**) NIH/3T3 cells viability with different treatments. Error bars indicate the SD (*n* = 5). (**c**) 24 h Scratch assay of NIH/3T3 cells with different treatments. (**d**) Quantification of scratch migration area. Error bars indicate the SD (*n* = 3). (**e**) Transwell assay of NIH/3T3 cells with different treatments. (**f**) Quantification of migration cells. Error bars indicate the SD (*n* = 3). (**g**) Tubule formation assay of HUVECs. Error bars indicate the SD (*n* = 3). (**h**) Quantitative analysis HUVEC total length. (**i**) Detection of ROS clearance with different treatments. (**j**) Quantification of mean fluorescence intensity (MFI). error bars indicate the SD (*n* = 3). Statistical differences were determined using an ANOVA with Bonferroni’s multiple comparison test (*p < 0.05, **p < 0.01, ***p < 0.001, ****p < 0.0001 compared to the control)

To investigate the effect of exo and exo^FGF 2^ ECM hydrogel, ECM/Cu^2+^ hydrogel, and exo^FGF 2^@ECM/Cu^2+^ hydrogel on cells, PKH-26 was used to label exo and exo^FGF 2^. Fluorescence micrographs (Fig. 2a) showed that exo, exo^FGF 2^ and exo^FGF 2^@ECM/Cu^2+^ hydrogels group could detected PKH-26-labelled exosomes (red fluorescence) were gradually internalized by NIH/3T3 cells (nucleus were stained with DAPI to blue fluorescence). By CCK-8 essay, all experimental groups increased the proliferation of NIH/3T3 cells, exo^FGF 2^ and exo^FGF 2^@ECM/Cu^2+^ hydrogels group significantly promoted cell proliferation (Fig. 2b). At the same time, both the wound healing and transwell results confirmed that exo^FGF 2^ and exo^FGF 2^@ECM/Cu^2+^ hydrogels group could significantly enhance the migration of NIH/3T3 cells. The migrating cells stimulated with exo^FGF 2^ and exo^FGF 2^@ECM/Cu^2+^ hydrogels possessed the similar migratory capacity (Fig. 2c, d, e, f). Then, the tube formation experiments demonstrated (*p* < 0.05, Fig. 2g, h) that the total vessel length was higher in the exo^FGF 2^ and exo^FGF 2^@ECM/Cu^2+^ hydrogels group. In addition, exo^FGF 2^ and exo^FGF 2^@ECM/Cu^2+^ hydrogels group displayed weak fluorescence intensity (FL), suggesting that exo^FGF 2^ successfully prevented cells from environmental oxidative stress (*p* < 0.0001, Fig. 2i, j). These findings indicated that exo^FGF 2^ exhibits more potent biological activity than exo. The exo^FGF 2^@ECM/Cu^2+^ hydrogel, through the sustained release of exo^FGF 2^, can elicit biological effects akin to exo^FGF 2^ itself. In contrast, the biological effects of ECM and ECM/Cu^2+^ were relatively weak.

### In vitro antimicrobial activity

**Fig. 3:**
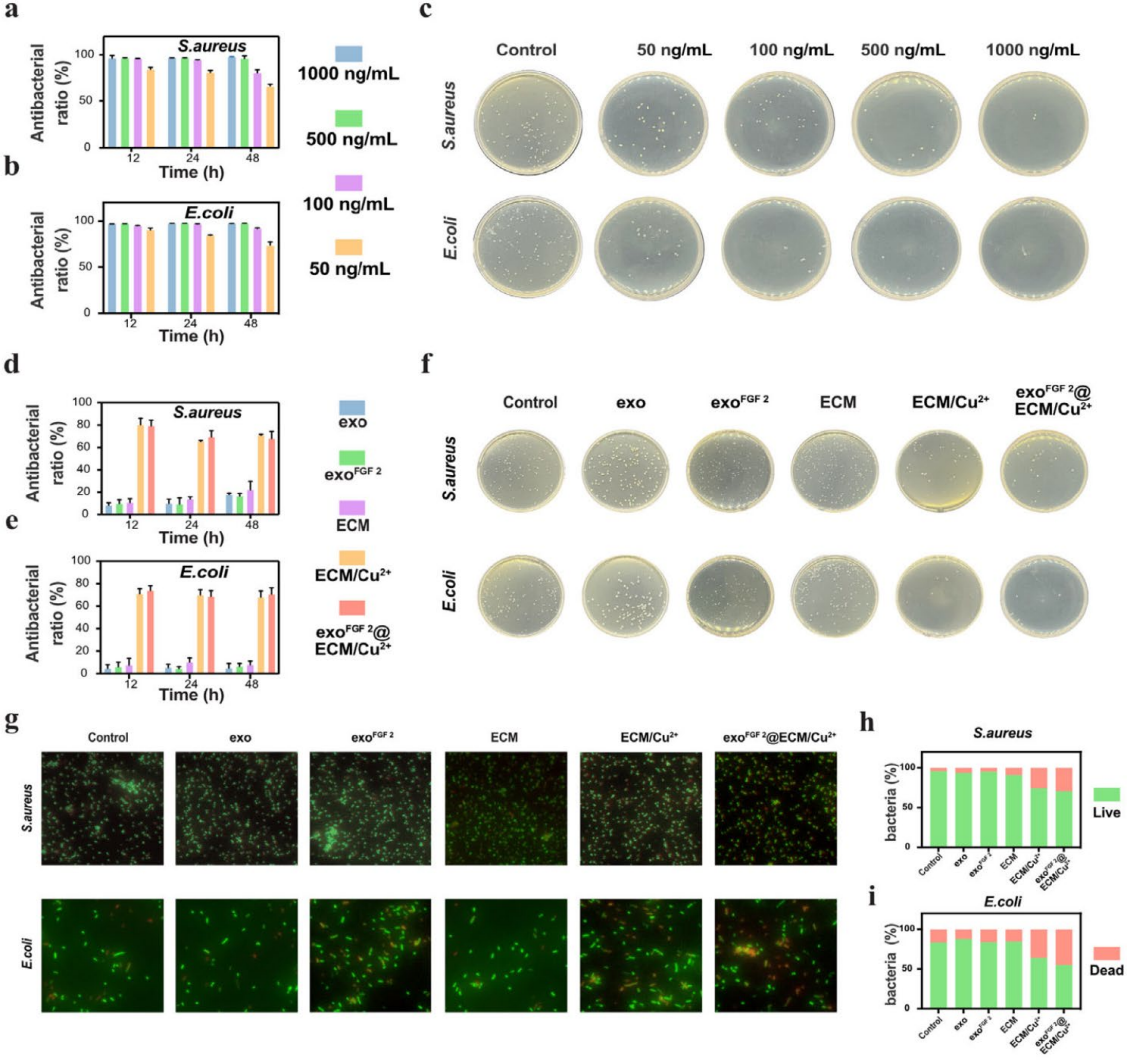
In vitro antimicrobial activity. The antibacterial rate of different concentrations of Cu^2+^ against (**a**) *S. aureus* and (**b**) *E. coli*. Error bars indicate the SD (*n* = 5). (**c**) Spread plate results of bacterial suspension co-cultured with different concentrations of Cu^2+^at 12 h. The antibacterial rate of different treatments against (**d**) *S. aureus* and (**e**) *E. coli*. Error bars indicate the SD (*n* = 5). (**f**) Spread plate results of bacterial suspension co-cultured with different treatments at 12 h. (**g**) Fluorescent images of live and dead bacterial staining of different treatments at 12 h, dead bacteria (red) and living bacteria (green). Quantification of fluorescence intensity of (**h**) *S. aureus* and (**i**) *E. coli*

Antimicrobial activity is one of the most important properties of hydrogel dressings during wound recovery. According to our experimental results, the antimicrobial effect of Cu^2+^ was directly proportional to its concentration (Fig. 3a, b, c), while the cytotoxicity was inversely proportional to its concentration (Fig. S3). Combining the results of these two experiments, we conclude that 100 ng/mL of copper ions can effectively inhibit bacterial growth without cytotoxicity. Therefore, in all subsequent experiments, the exo^FGF 2^@ECM/Cu^2+^ hydrogels were referred to as a hydrogel containing 100 ng/mL of copper ions.

To further evaluate the antimicrobial capacity of exo, exo^FGF 2^, ECM hydrogels ECM/Cu^2+^ hydrogels and exo^FGF 2^@ECM/Cu^2+^ hydrogels bacterial co-cultures were performed. These results suggest that Cu^2+^ released from ECM/Cu^2+^ hydrogels and exo^FGF 2^@ECM/Cu^2+^ hydrogels could effectively inhibited bacterial proliferation (Fig. 3d, e,f). Then, bacteria was tagged with DMAO and EthD-III fluorescent to further investigate whether exo^FGF 2^@ECM/Cu^2+^ hydrogels and ECM/Cu^2+^ hydrogels induce mortality of bacteria. The staining process was followed by fluorescence microscope (Leica, Germany) observation. The results of the live-dead staining of bacteria can be visualized by observing a higher percentage of dead bacteria in the hydrogel containing copper ions (Fig. 3g, h, i).

### In vivo and in vitro biocompatibility

**Fig. 4:**
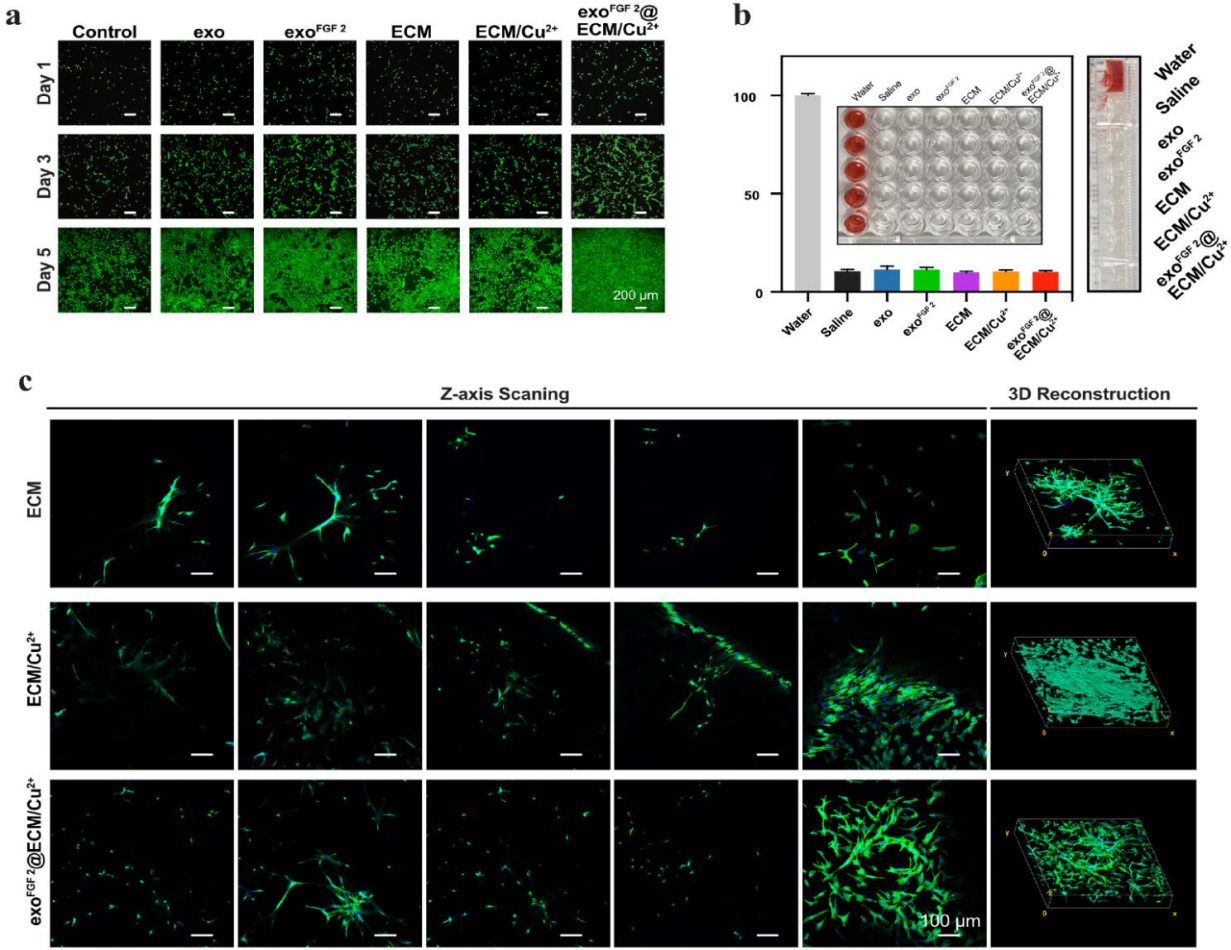
Biocompatibility evaluations of exo, exo^FGF 2^, ECM hydrogels, ECM/Cu^2+^hydrogels and exo^FGF 2^@ECM/Cu^2+^ hydrogels. (**a**) Representative fluorescence microscopy images of NIH/3T3 cells stained by live/dead staining assay on Day 1, Day 3 and Day 5. (**b**) Hemolysis ratio and lateral hemolysis photos. Inset: Top view of the 96-well plates before measuring OD values. Error bars indicate the SD (*n* = 5). (**c**) CLSM of NIH/3T3 cells on ECM hydrogels, ECM/Cu^2+^hydrogels and exo^FGF 2^@ECM/Cu^2+^ hydrogels after 5 days post-seeding. F-actin (green), nuclei (blue)

The biocompatibility of NIH/3T3 cells were further assessed after co-culturing them with different EVs and different ionic extracts of hydrogels. These results indicated that the samples were non-toxic to cells (Fig. 4a). And there was no significant haemolysis detected in the erythrocyte haemolysis test, indicating that the hydrogels were benignly haemocompatible (Fig. 4b). When co-cultured with ECM hydrogels, ECM/Cu^2+^ hydrogels and exo^FGF 2^@ECM/Cu^2+^ hydrogels, NIH/3T3 cells showed desirable plump cytoplasm, well-defined nuclei and typical spindle shape (Fig. 4c). These results indicated that this hydrogel are cytocompatible. Finally, EVs and hydrogel samples were implanted subcutaneously in mice for 10 days (Fig. S4). No damage to major organs (heart, liver, spleen, lungs and kidneys) were detected by hematoxylin and eosin (H&E) staining.

### In vivo wound closure measurements in a full-thickness skin defect model

**Fig. 5:**
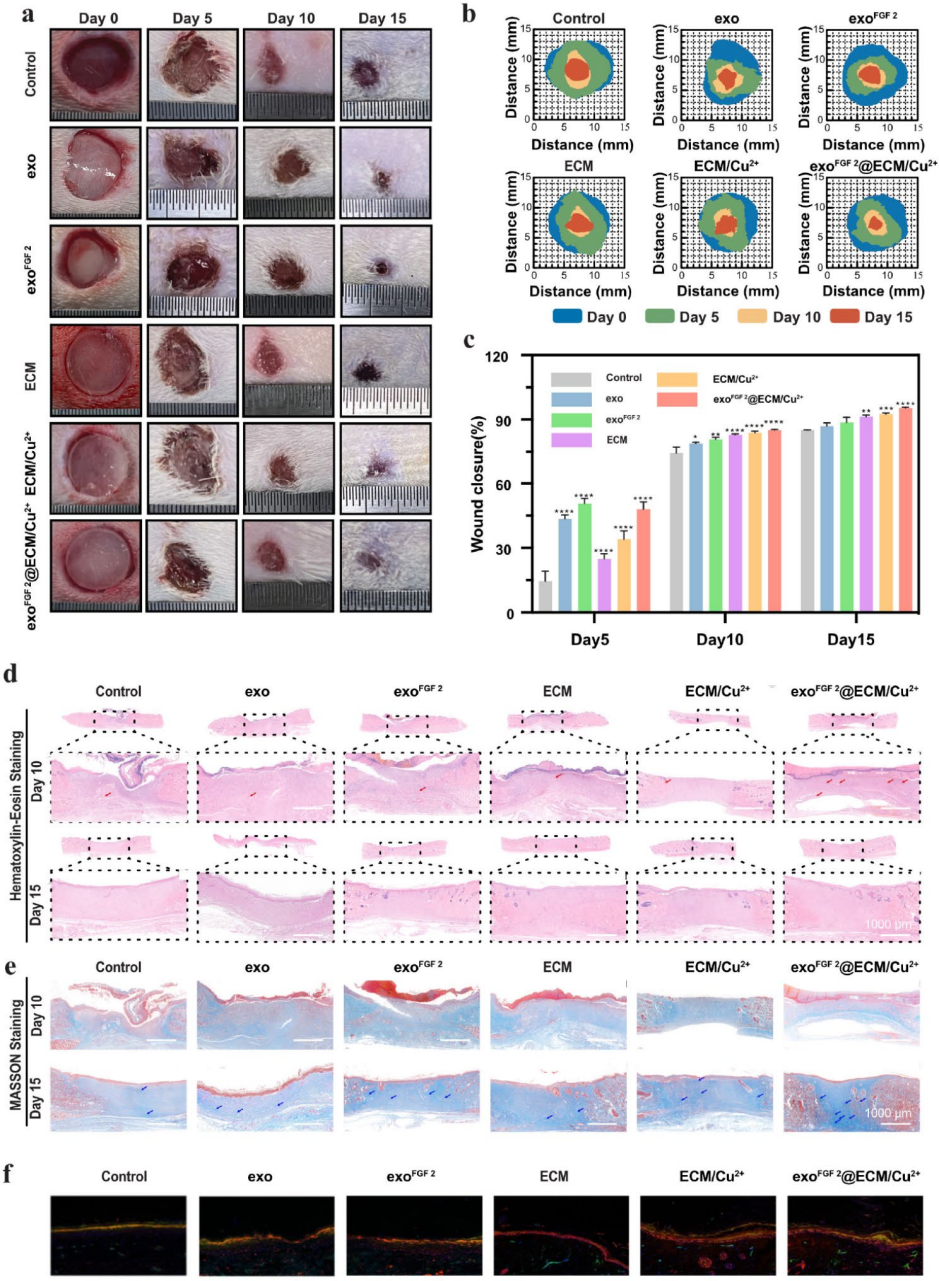
In vivo wound healing evaluation. (**a**) Digital images of full-thickness skin defects of SD rats with different treatments on Day 0, 5,10 and 15. (**b**) Schematic diagram of the skin repair process in SD rats. (**c**) Wound closure percentages at different time points. Error bars indicate the SD (*n* = 3). (**d**) H&E and (**e**) Masson staining of wounds on Day10 and 15. Scale bar is 100 μm. (**f**) Immunofluorescence staining of the regenerated wound tissue with VEGF (red) and CD34 (green) on Day15. Statistical differences were determined using an ANOVA with Bonferroni’s multiple comparison test (*p < 0.05, **p < 0.01, ***p < 0.001, ****p < 0.0001 compared to the control)

The wound-healing properties of EVs and hydrogels were investigated in a full-thickness skin defect model of rats. Wound images were collected on Day 0, Day 5; Day 10 and Day 15 for quantitatively assess the wound healing effect of different groups. As in Fig. 5a, even though all incisions closed over time, the experimental group had a substantially higher percentage of wound closure than the control group. exo^FGF 2^ group and exo^FGF 2^@ECM/Cu^2+^ group had almost identical therapeutic effect on Day 5. In the exo^FGF 2^@ECM/Cu^2+^ group, in particular, there was a significant narrowing of the incisions during each period of the healing process with a wound contraction ratio higher than 97.7% on Day 15. For further visualisation of the variations of wound area during wound healing, traces of wound bed closures have been drawn by Image J (Fig. 5b, c). It can be summarized that exo^FGF 2^ have a good therapeutic effect in the early stages, and its biological activity fades over time. However, exo^FGF 2^@ECM/Cu^2+^ hydrogel can achieve long-term effective therapeutic effects by slowly releasing exo^FGF 2^.

Wound tissues were collected on Day 10 and Day 15 for Haematoxylin and eosin (H&E) and Masson trichrome staining to evaluate wound repair efficiencies from a histological perspective. As depicted in Fig. 5d, compared with other groups, the exo^FGF 2^ group and exo^FGF 2^@ECM/Cu^2+^ group showed the higher number of newly formed blood vessels on Day 10, which can be attributed to the components of therapeutic exo^FGF 2^. Although all groups had constituted the fundamental structure of the epithelium and dermis on Day 15, the control group tissue was still in the healing process, and no hair follicles were found. Nonetheless, the ECM and exo^FGF 2^@ECM/Cu^2+^ hydrogel groups exhibited more regular epithelium. Furthermore, the entire H&E staining results of the wounds indicate that a thicker epidermal layer and a thicker granulation tissue were observed in the groups treated by exo^FGF 2^@ECM/Cu^2+^ hydrogels Fig. 5d, suggesting the good wound healing effect, including the improvement of re-epithelialization process and dermal reconstruction (Fig. S5a, b). Collagen is one of the vital constituents for the structure and function of healthy skin and also a very significant sign for the remodelling of injured skin tissue. The deposition of collagen fiber in all groups were distributed sparsely on Day 10. On Day 15, the exo^FGF 2^@ECM/Cu^2+^ group showed denser collagen fiber deposition, as shown in blue staining and blue arrow in Fig. 5e.

To further evaluate the proliferation of blood vessels in these groups, immunohistochemical staining was used on Day 15 to examine the expression of VEGF as a tissue enrichment. VEGF is mainly expressed in vascular endothelial cells, fibroblasts, and M2 macrophages, which can promote angiogenesis and improve the supply of nutrients and oxygen in the wound site to accelerate wound healing. The exo^FGF 2^@ECM/Cu^2+^ group expressed more VEGF on Day 15 than the other groups(Fig. 5f, Fig. S5c). CD34-positive nuclei are indicative of the process of re-epithelialization. Only a small number of CD34-positive nucleus were observed in wound samples from the control group, whereas in the ECM and exo^FGF 2^@ECM/Cu^2+^ hydrogel groups, they were gradually distributed in the newly formed tissue filling the wound center. The ECM group displayed greater CD34 marker expression than the control group, with exo^FGF 2^@ECM/Cu^2+^ exhibiting the highest levels of all groups (Fig. S5d)

## Discussion

Recent studies have found that stem cells from various tissues are capable of regenerative repair through the formation of secretory extracellular vesicles [20, 21]. These vesicles regulate cell proliferation, migration, and multilineage differentiation by binding to receptor cells [22–24]. Furthermore, stem cell extracellular vesicles can transport nucleic acids, proteins, and small-molecule medications which results in enhanced stability, bioavailability, and sustained release of drugs [25, 26]. According to Vashisht et al., Compared to free curcumin, curcumin encapsulated in extracellular vesicles exhibits greater stability [27]. This encapsulation safeguards curcumin against the digestive environment and permits it to traverse the intestinal barrier. Prior research has demonstrated that FGF 2 is an exceptionally efficacious therapeutic agent for pressure ulcers [28, 29]. Additionally, several studies have demonstrated that bFGF-loaded hydrogels can hasten the healing of wounds [14, 30, 31]. However, the limited bioavailability and poor in vitro stability of bFGF have greatly hindered its widespread clinical use [32]. Extracellular vesicles act as transport mediators for FGF 2, improving its bioavailability and stability, facilitating its transport to cells, and enhancing its biological activity. In this study, both exo and exo^FGF 2^ substantially increased cell proliferation and exo^FGF 2^ was more optimal for tissue repair due to its enhanced pro-proliferative, vasculogenic, and anti-oxidative free radical properties.

In many clinical applications, extracellular matrix (ECM) bioscaffolds are used to remodel tissue structure and function [33]. ECM hydrogels are formed by the self-assembly of collagen, which is partially controlled by glycosaminoglycans, proteoglycans and ECM proteins [34]. The inherent biochemical properties of the proteins have an impact on the gel formation effect [35]. Therefore, effective cell removal while maintaining the integrity of the extracellular matrix (ECM) is critical to the gel formation process. A dry weight DNA concentration of less than 50 ng/mg, a DNA fragment length of less than 200 bp, or the absence of visible nuclear material in DAPI or H&E stains are the minimum requirements for successful decellularization [36, 37]. In this study, the test results indicate that nearly all cell-associated components of ECM were eliminated, thereby preventing immune reactions induced by ECM. Chemical bonding between metal ions and ligands can also help ECM proteins construct three-dimensional network structures. The addition of trace metal ions to the hydrogels enhance its stability and imparts antimicrobial properties [38, 39]. The potential of copper ions to promote cellular proliferation and possess antibacterial properties has been well documented [40]. In this study, the introduction of copper ions resulted in enhanced stability and antibacterial properties for the ECM hydrogels. The degradation results of this experiment showed that exo^FGF 2^@ECM/Cu^2+^ containing copper ions degraded more slowly. This suggests the structural integrity of the hydrogel has been significantly enhanced. Nevertheless, the presence of copper ions may also affect the activity of collagenase, resulting in a corresponding reduction in the hydrogel’s degradation rate. By slowly releasing copper ions over a period of 12 days, the hydrogel sustained its bacteriostatic effect while preventing cytotoxicity that could arise from high concentrations of copper ions. These features guarantee an efficient wound-healing process.

The considerable capacity of exo^FGF 2^@ECM/Cu^2+^ hydrogel to accelerate wound healing was further demonstrated through a 15-day in vivo study using a full-thickness skin defect model. Compared to other groups, H&E staining showed the most significant epithelialization in the lesions of the exo^FGF 2^@ECM/Cu^2+^ group. CD34/VEGF immunofluorescence staining confirmed that the exo^FGF 2^@ECM/Cu^2+^ group exhibited the highest vascular density on Day 15. On Day 15, VEGF immunohistochemical staining indicated that the majority of vessels in the exo^FGF 2^@ECM/Cu^2 +^ group had matured and grown more than other groups. Conversely, nuclei positive for CD34 exhibited the most pronounced expression, indicating an enhanced capacity to stimulate cellular proliferation. Furthermore, Masson trichrome staining showed greater collagen accumulation in the exo^FGF 2^@ECM/Cu^2+^ group. On Day 15, dermal collagen was more uniform in the exo^FGF 2^@ECM/Cu^2+^ group than in the control group or in individuals with normal skin. These findings indicate that exo^FGF 2^@ECM/Cu^2+^ hydrogels have the potential to stimulate angiogenesis, collagen deposition, and re-epithelialization in vivo.

## Conclusion

In conclusion, exo^FGF 2^ loaded dECM/Cu^2+^ hydrogels were successfully fabricated. exo^FGF 2^@ECM/Cu^2+^ hydrogels have many desirable properties including biodegradability, biocompatibility, antibacterial and hemostatic activity as well as a good wound healing potential. In vitro, exo^FGF 2^@ECM/Cu^2+^ hydrogels significantly improved the proliferation and migration of NIH/3T3 cells, the scavenging of oxygen free radicals, and the angiogenesis of HUVECs. Further, in vivo, studies showed that in a normal animal wound model, exo^FGF 2^@ECM/Cu^2+^ hydrogels enhanced cell proliferation and migration, angiogenesis and collagen deposition, and reduced inflammatory responses. These results suggest that a new strategy was provided for more effective treatment of normal wound healing by incorporating copper ions and exosomes into ECM hydrogels. Despite our findings, the limitations of this study should be noted. For example, it is critical to determine the specific activity of exosome components in wound healing and skin regeneration, but our study did not fully address this issue. Going forward, researchers should focus on elucidating these exact components and their effects on wound healing. This approach will enhance the potential for clinical application of these findings.

### Electronic supplementary material

Below is the link to the electronic supplementary material.

Supplementary Material 1

## Data Availability

No datasets were generated or analysed during the current study.

## Abbreviations

ECM: Extracellular matrix
dECM: decellularized extracellular matrix
FGF 2: Fibroblast growth factor 2
exo: Human umbilical cord mesenchymal stem cell derived exosome
exo^FGF 2^: Growth factor 2 containing exosom;
EVs: exo and exo^FGF 2^
hUCMSC: human umbilical cord mesenchymal stem cell
HUVEC: Human umbilical vein endothelial cell
TEM: Transmission electron microscopy
CLSM: Confocal laser scanning microscope
