# Supplementary material for: Extracellular matrix hydrogels with fibroblast growth factor 2 containing exosomes for reconstructing skin microstructures
Source: J Nanobiotechnology. 2024 Jul 26;22:438. doi: 10.1186/s12951-024-02718-8 (PMC11282598; doi:10.1186/s12951-024-02718-8)
Supplement: Supplementary file 1 — Supplementary Material 1 [file 12951_2024_2718_MOESM1_ESM.docx]

| 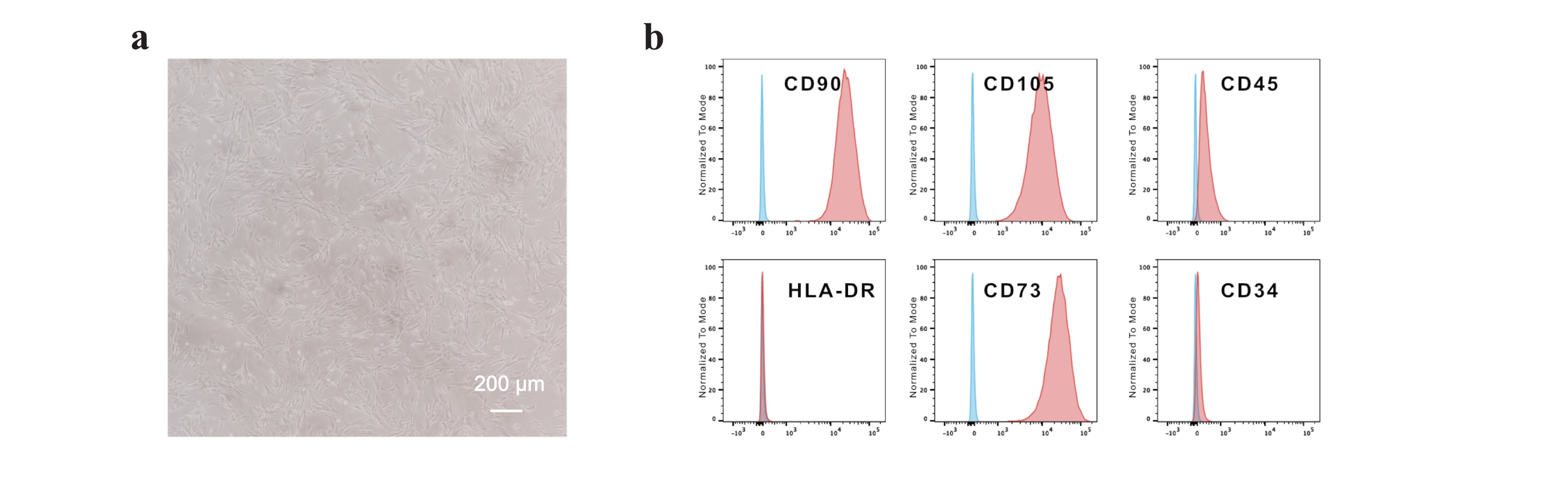 |
| --- |
| **Fig. S1.** a) hUCMSCs morphology under light microscopy. b) Characteristic surface markers of hUMSCs evaluated by flow cytometry. The blue curves represent isotype controls and the red curves represent the surface markers. |

| 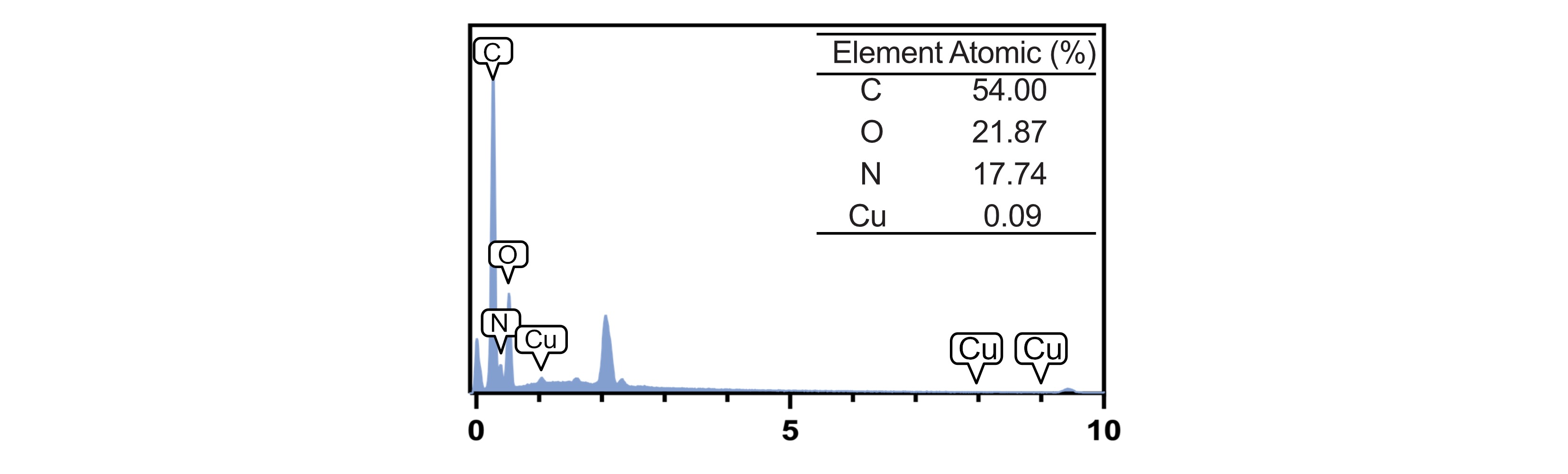 |
| --- |
| **Fig. S2.** The Energy-dispersive X-ray spectroscopy (EDS) data shows the semi-quantitative composition of each element in exo^FGF 2^@ECM/Cu^2+^ scaffolds. |

| 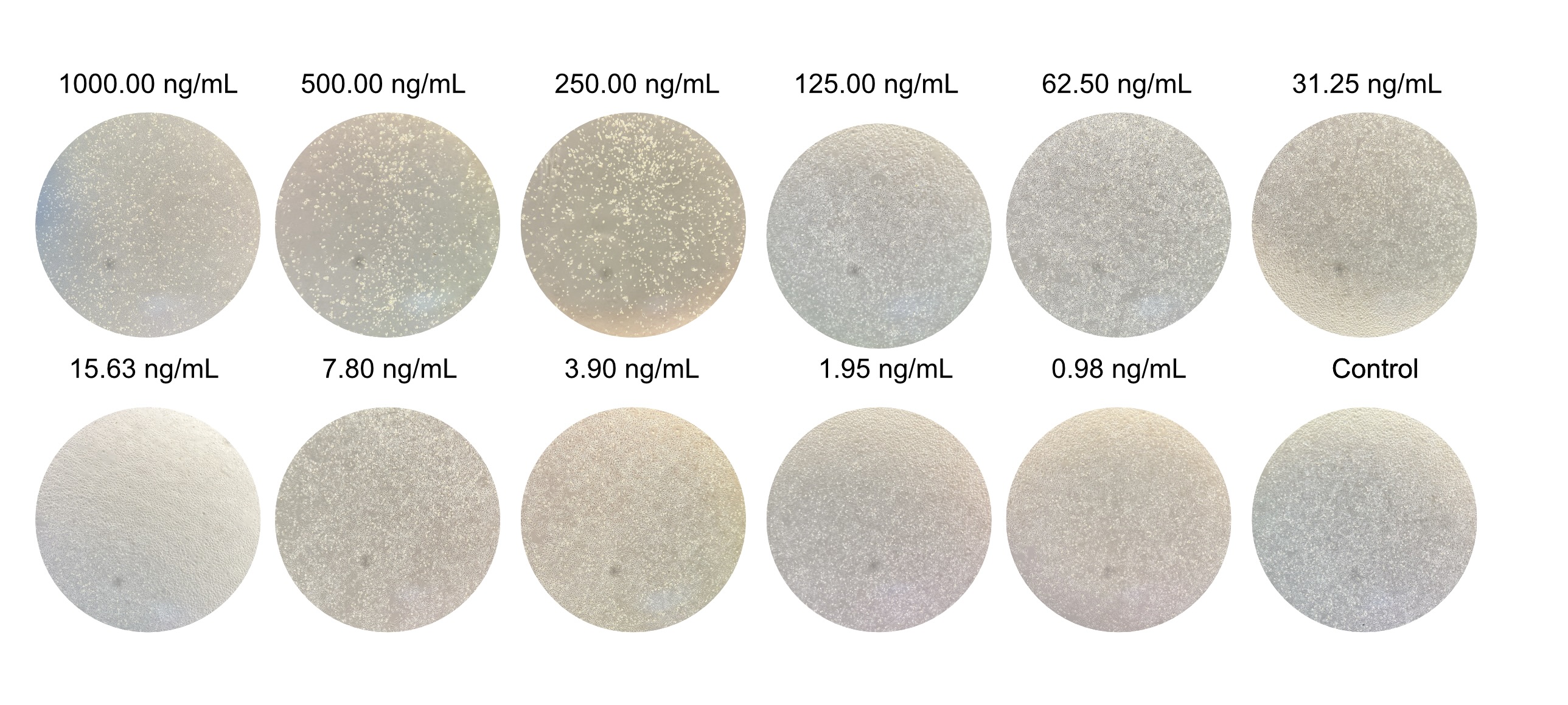 |
| --- |
| **Fig. S3.** Light microscopic morphology of NIH/3T3 cells after 12 h of co-culture with different concentrations of copper ions. |

| 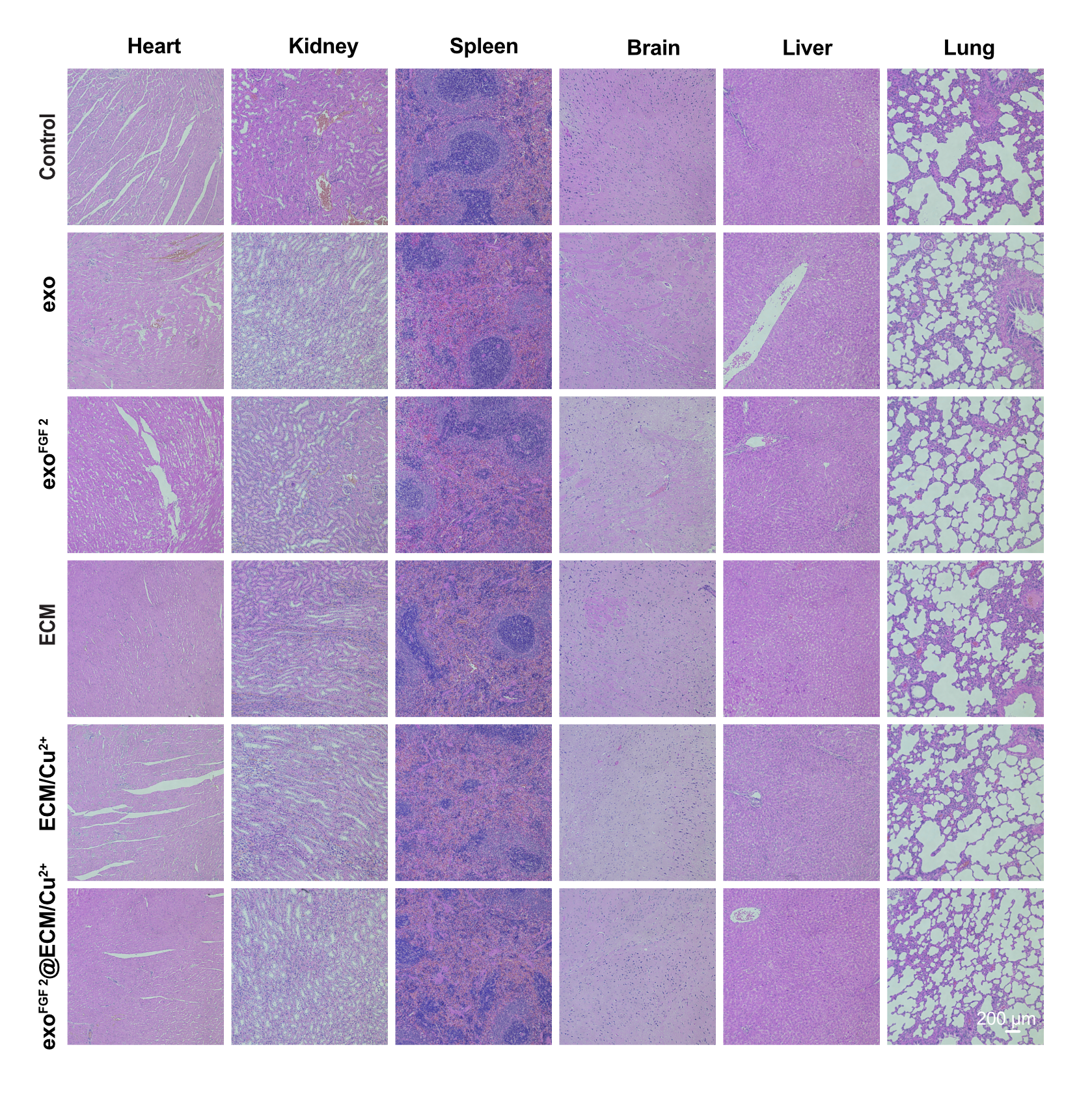 |
| --- |
| **Fig. S4.** H&E images of various organs from SD mice after a 14-day treatment with PBS, exo, exo^FGF 2^, ECM hydrogels ECM/Cu^2+^ hydrogels and exo^FGF 2^@ECM/Cu^2+^ hydrogels in rate. |


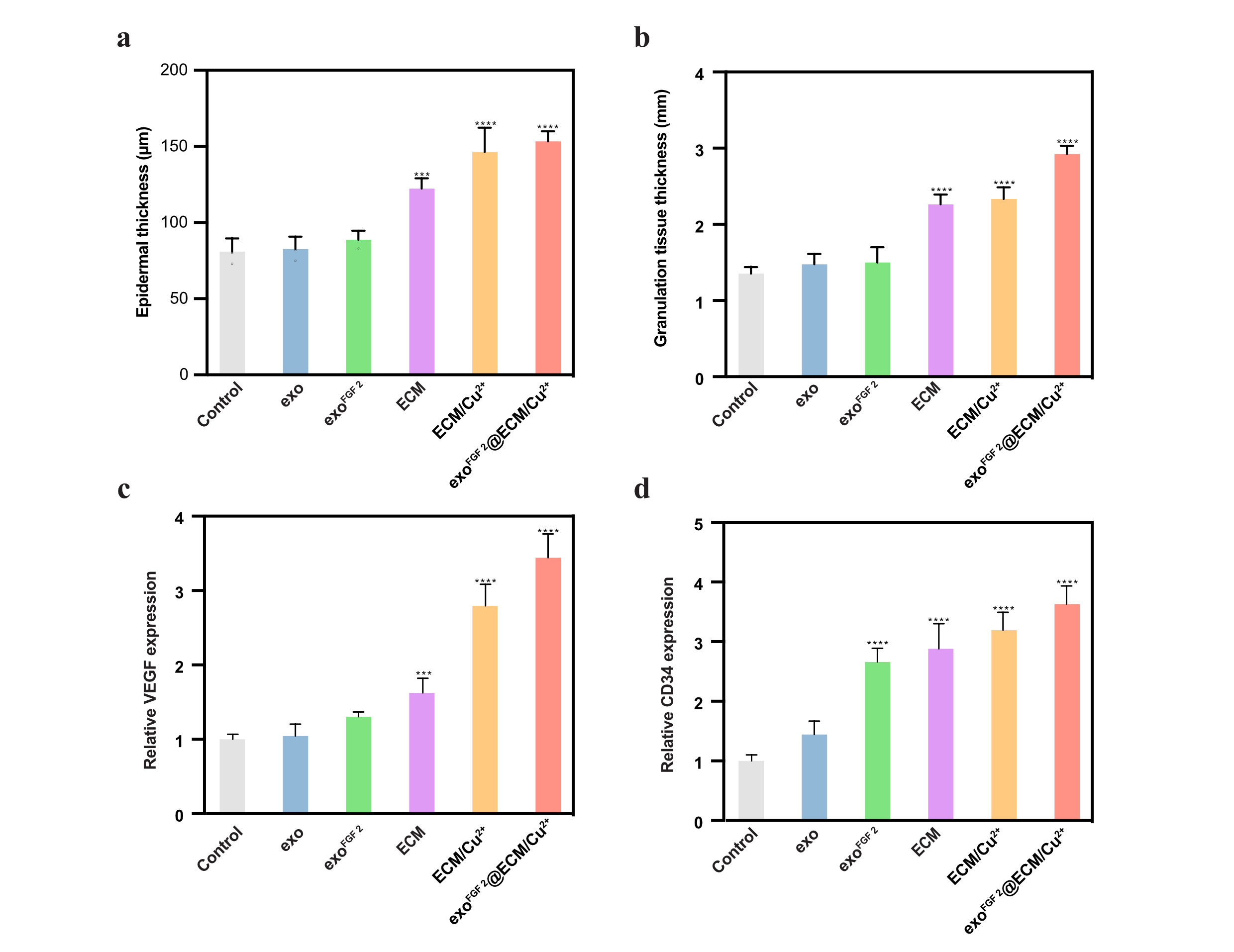


**Fig. S5.** a) The thicknesses of the epidermal layer. b) The thicknesses of the granulation tissue. Quantification of c)VEGF and d)CD34 expression. Error bars indicate the SD (*n* = 3). Statistical differences were determined using an ANOVA with Bonferroni's multiple comparison test (**p* < 0.05, ***p* < 0.01, ****p* < 0.001, *****p* < 0.0001 compared to the control).


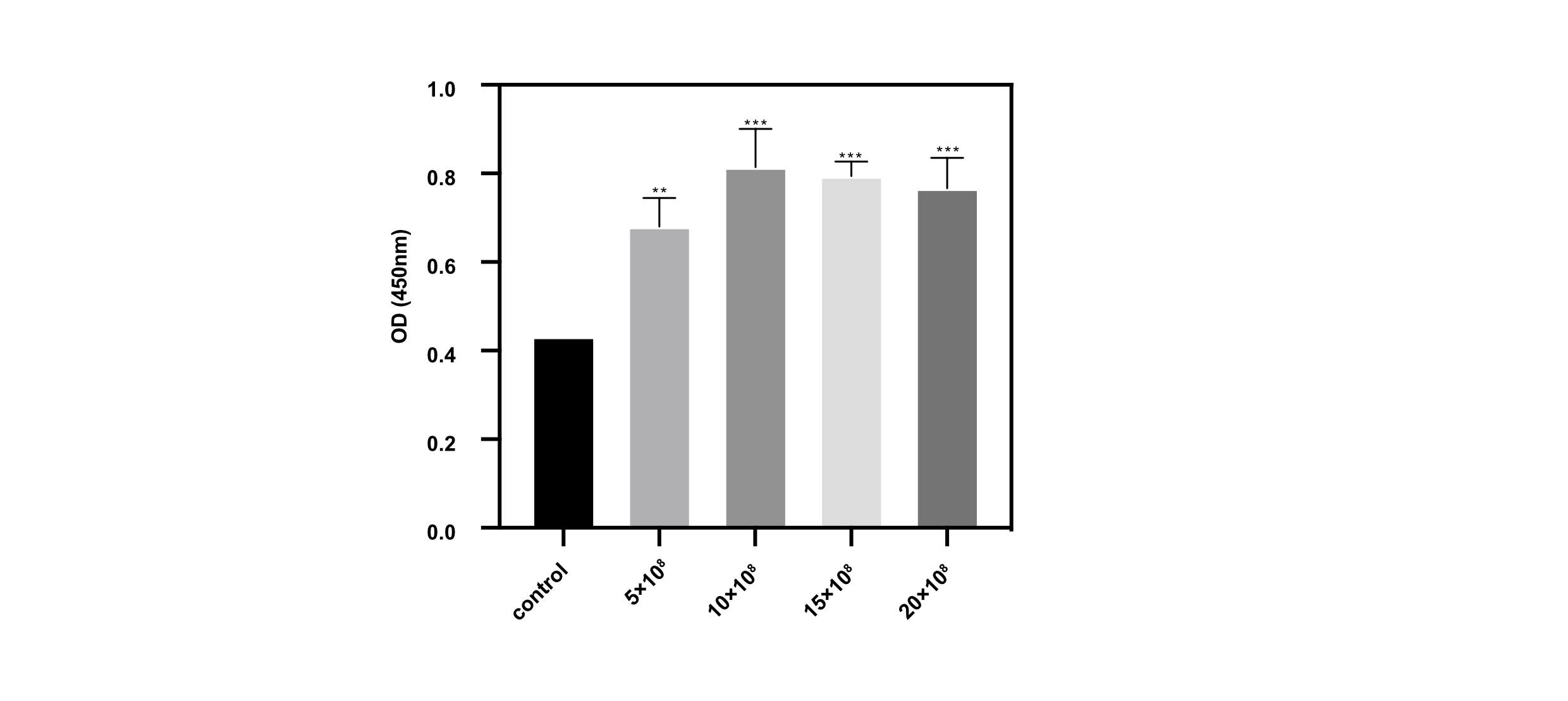


**Fig. S6.** NIH/3T3 cells viability with different treatments. Error bars indicate the SD (*n* = 5). Statistical differences were determined using an ANOVA with Bonferroni's multiple comparison test (**p* < 0.05, ***p* < 0.01, ****p* < 0.001, *****p* < 0.0001 compared to the control).


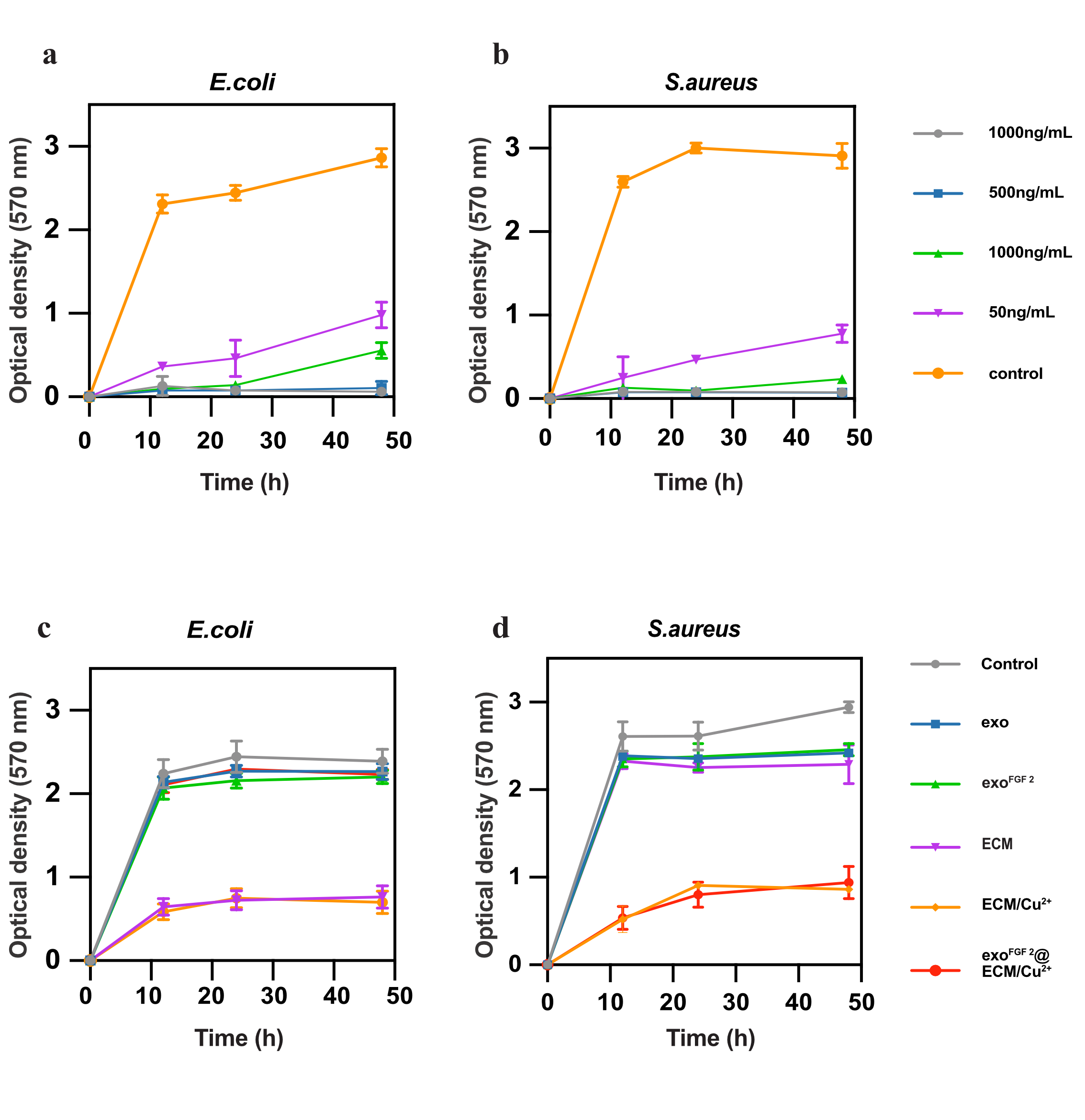


**Fig. S7.** Antibacterial activity of Cu^2+^ and exo^FGF 2^@ECM/Cu^2+^ hydrogels. Bacterial growth curves of a) *E. coli*. and b) *S. aureus* with different concentrations of Cu^2+^ at different times. Error bars indicate the SD (*n =* 3). Bacterial growth curves of c) *E. coli*. and d) *S. aureus* with exo, exo^FGF 2^ ,ECM hydrogels, ECM/Cu^2+^ hydrogels, exo^FGF 2^@ECM/Cu^2+^ hydrogels. Error bars indicate the SD (*n =* 3).
